# Rotating opportunistic prevalence audit: A new pragmatic audit method is effective at identifying misuse of ciprofloxacin in a large academic hospital

**DOI:** 10.1017/ice.2022.78

**Published:** 2023-06

**Authors:** Justin Z. Chen, David A. Waldner, Lynora M. Saxinger

**Affiliations:** Division of Infectious Diseases, Department of Medicine, Faculty of Medicine & Dentistry, University of Alberta, Edmonton, Alberta, Canada

## Abstract

The rotating opportunistic prevalence audit (ROPA) methodology is described with ciprofloxacin prescriptions in adults hospitalized at a tertiary-care center. Prescription appropriateness was assessed. ROPA captured 82% of all ciprofloxacin prescriptions; 69% were inappropriate. ROPA is feasible and may overcome resource barriers of prospective audit and feedback.

The Infectious Diseases Society of America (IDSA) guidelines for implementing an antimicrobial stewardship programs (ASPs) recommend prospective audit and feedback (PAF) as a core strategy.^1^ Many PAF interventions target a unit, program, or population rather than broad-based implementation due to resource limitations.^2–5^

The University of Alberta Hospital (UAH) is a 700-bed, tertiary-care center in Edmonton, Canada, with an ASP comprising 0.45 full-time equivalent (FTE) ASP physicians and 0.8 FTE ASP pharmacists. The PAF of 6 broad-spectrum antibiotics (meropenem, imipenem, ertapenem, daptomycin, linezolid, and tigecycline) is a core initiative.

Fluoroquinolones are a common ASP priority due to antimicrobial resistance in Enterobacterales^6^ and adverse effects.^7^ Ciprofloxacin was the second most utilized antimicrobial at our center in the years preceding this study. The high volume of prescriptions previously precluded appropriateness assessments.

The rotating opportunistic prevalence audit (ROPA) methodology was conceptualized to efficiently assess high-volume antimicrobial prescriptions by prioritizing units with the highest number of unaudited active orders for the target antimicrobial(s), then mapping a rotation on lower-use units by location to maximize efficiency and sampling.

The primary objective of this study was to assess the feasibility and sampling completeness of the ROPA method using ciprofloxacin. The secondary objective was to assess the utility of ROPA audit appropriateness data in identifying prescribing patterns, thus informing future interventions.

## Methods

The inpatient pharmacy system (Cerner Millennium, Cerner, Kansas City, MO) was used to identify new or active ciprofloxacin prescriptions that were added to a rolling list organized by unit. Intravenous and oral ciprofloxacin prescriptions in patients aged ≥18 years who were hospitalized at the UAH were eligible for ROPA. Prescriptions were excluded from ROPA if it was a 1-time dose, if it was discontinued prior to assessment, or if the patient was discharged prior to audit. Units were prioritized for in-person audit by the number of unaudited prescriptions over the past 3 days. Further audit capacity was assigned by the elapsed time since a unit was last audited and by unit location favoring adjacent units. ROPA was performed over 10 consecutive weekdays in April 2018. Paper-chart audit and data entry were allotted 4 hours daily. This resulted in a focus on high-use units with sampling across many units. In this process pilot, contemporaneous prescriber feedback was not provided.

Appropriateness was assessed against institutional prescribing guidelines (ie, Bugs & Drugs) and/or AHS Provincial Drug Formulary Usage Guidelines (Appendix 1 online) in domains of indication, spectrum, route, dose, and allergy/intolerance history. An ID subspecialty resident (D.A.W.) and an ID/ASP physician (L.M.S.) performed the audits with dual review of appropriateness assessment. Discordant assessments were resolved by consensus.

The primary outcome was the proportion of auditable ciprofloxacin prescriptions captured by the ROPA method during the audit period. The secondary outcome was the percentage of audited prescriptions assessed as appropriate.

Data were prospectively collected and descriptive analyses were performed. The University of Alberta Research Ethics Board (no. Pro00107975) granted ethics approval.

## Results

In total, 91 unique ciprofloxacin prescriptions met the inclusion criteria over the 2-week period; no repeat or duplicate prescriptions were detected. The mean patient age was 64 years and 59% of prescriptions were for female patients. The ROPA method captured 75 (82%) of 91 eligible ciprofloxacin prescriptions; the remainder were not audited due to time constraints. Of 75 prescriptions, 25 (33%) were intravenous and 31 (41%) were for empiric indications. The most commonly recorded indication was urinary tract infection (24 of 75, 32%).

The average number of completed audits was 8 per day (range, 4–13). Of 75 prescriptions, 43 (57%) were audited within 1 day of prescribing, 72% were audited within 3 days, and 87% were audited within 7 days. Of 24 inpatient programs, 18 were audited at least once over 2 weeks. The highest-use programs were medicine (61%), surgery (25%), and critical care (12%). Of the 75 prescriptions, attending physicians wrote 31 prescriptions (41%) and 11 (35%) were assessed as appropriate. Resident physicians wrote 38 prescriptions (51%) and 12 (32%) were assessed as appropriate. Moreover, 71% of orders were made between 09:00 and 17:00, and 16 (30%) of these were assessed as appropriate. Similarly, 7 (32%) of 22 after-hours prescriptions were assessed as appropriate.

Overall, 52 (69%) of 75 audited prescriptions were assessed as inappropriate. Also, 21 (40%) of 52 had inappropriate indication: 12 for asymptomatic bacteriuria, 6 for postoperative prophylaxis, and 3 without evidence of infection. Of these 52 prescriptions, 17 were inappropriate due to inadequate spectrum, 6 were inappropriate due to dose, 7 were inappropriate due to administration route, and 1 was inappropriate due to documented fluoroquinolone-associated tendinopathy.

In 20 cases for which ciprofloxacin was used to treat *Pseudomonas* spp or Enterobacterales classically producing an AmpC β-lactamase, 83% were assessed as appropriate.

## Discussion

Broadly implemented daily PAF can be resource intensive.^1^ Therefore, implementation strategies often focus on specific units, programs, or populations. However, this approach limits the potential impact of broader engagement, exposure, and education. The ROPA strategy frees auditors from strict unit or program inclusion and the expectation of 100% capture. ROPA optimizes on-unit audit time and representation in sampling by prioritizing volume and unit location (with cluster sampling of adjacent units for efficiency). ROPA, performed for 4 hours daily, captured 82% of all ciprofloxacin prescriptions despite our center averaging ∼100 World Health Organization (WHO)–defined daily doses of ciprofloxacin per 1,000 patient days annually for 3 years preceding the study, consistent with other centers.^8,9^

Transit time between units is an underappreciated efficiency consideration.^10^ With ROPA, transit time is minimized because the auditor typically reviewed prescriptions in only 1–3 units per day. Nevertheless, the average number of audits per day was high, and most inpatient programs were covered during the 2-week study period. This methodology allowed identification and categorization of inappropriate ciprofloxacin use at our center. Inappropriate use was identified in 69% of prescriptions audited. These data were used to design a subsequent targeted ASP intervention.

This study had several limitations. This pilot study did not include prescriber feedback; thus, the additional time required to generate feedback and evaluate prescriber response would likely affect the number of audits conducted per unit time. The overall effectiveness of ROPA-based PAF with intermittent, unscheduled audits versus constant PAF in improving appropriateness requires comparative study. Further study is required to determine whether other ASP provider roles would yield similar results. However, we hypothesize that any ASP team composition could use ROPA because the main difference from constant PAF is an efficiency-maximizing sampling strategy that balances a small sacrifice in data completeness with a potentially broader ASP presence in high-use areas. Although long-term sustainability is unknown, thoughtfully designed ROPA interventions represent a sustainable and flexible method of assessing the use of priority antimicrobials within a constrained period. For example, a rotating series of antimicrobial ROPAs for 2–4 weeks each (allowing comparisons over time) or augmenting pre-existing ASP reach with intermittent projects (assessing a single agent during trainee rotations) could be explored.

In conclusion, ROPA was able to capture the majority of new ciprofloxacin prescriptions at our center for appropriateness audit over a short study period in a time-efficient manner, and this strategy allowed useful description of appropriateness patterns. Further assessment of the efficiency and effectiveness of ROPA with integrated prescriber feedback is required. ROPA may be a valuable audit strategy in resource-constrained ASPs using traditional unit-based audits and may allow targeted assessment of additional antimicrobials to identify ASP priorities.

## Supplementary material

For supplementary material accompanying this paper visit https://doi.org/10.1017/ice.2022.78. click here to view supplementary material
