# Supplementary material for: Rotating opportunistic prevalence audit: A new pragmatic audit method is effective at identifying misuse of ciprofloxacin in a large academic hospital
Source: Infect Control Hosp Epidemiol. 2022 Apr 12;44(6):951–3. doi: 10.1017/ice.2022.78 (PMC10262160; doi:10.1017/ice.2022.78)
Supplement: Supplementary file 1 [file S0899823X22000782sup001.docx]

**Appendix 1**

**Alberta Health Services Provincial Drug Formulary Usage Guidelines for ciprofloxacin**

1. Therapy of community/hospital acquired Gram negative infections.
2. Therapy of *Pseudomonas aeruginosa* infections in patients with ß-lactam allergy or unable to take IV antibiotics.
3. Therapy/stepdown therapy of polymicrobial infections in combination with clindamycin or metronidazole.
4. Therapy of genitourinary tract infections.
5. Prophylaxis of transurethral surgical procedures.
6. Therapy of suspected bacterial gastroenteritis (Most cases of diarrhea are self-limiting and do not require antimicrobial therapy).
7. Prophylaxis of adult contacts of cases of invasive meningococcal disease.
8. Empiric therapy in low risk febrile neutropenic patients (temp. = 38.3ºC, absolute neutrophil count < 0.5 x 109/L) in combination with amoxicillin-clavulanate.
9. Prophylaxis in patients at risk of Gram negative infections (cirrhosis, neutropenia).
